# The Impact of Combining a Low-Tube Voltage Acquisition with Iterative Reconstruction on Total Iodine Dose in Coronary CT Angiography

**DOI:** 10.1155/2017/2476171

**Published:** 2017-05-23

**Authors:** Toon Van Cauteren, Gert Van Gompel, Kaoru Tanaka, Douwe E. Verdries, Dries Belsack, Koenraad H. Nieboer, Inneke Willekens, Paul Evans, Sven Macholl, Guy Verfaillie, Steven Droogmans, Johan de Mey, Nico Buls

**Affiliations:** ^1^Radiology, Vrije Universiteit Brussel (VUB), Universitair Ziekenhuis Brussel (UZ Brussel), Laarbeeklaan 101, 1090 Brussels, Belgium; ^2^Imaging R&D, GE Healthcare Life Sciences, The Grove Centre, Amersham, Buckinghamshire, UK

## Abstract

**Objectives:**

To assess the impact of combining low-tube voltage acquisition with iterative reconstruction (IR) techniques on the iodine dose in coronary CTA.

**Methods:**

Three minipigs underwent CCTA to compare a standard of care protocol with two alternative study protocols combining low-tube voltage and low iodine dose with IR. Image quality was evaluated objectively by the CT value, signal-to-noise ratio (SNR), and contrast-to-noise ratio (CNR) in the main coronary arteries and aorta and subjectively by expert reading. Statistics were performed by Mann–Whitney *U* test and Chi-square analysis.

**Results:**

Despite reduced iodine dose, both study protocols maintained CT values, SNR, and CNR compared to the standard of care protocol. Expert readings confirmed these findings; all scans were perceived to be of at least diagnostically acceptable quality on all evaluated parameters allowing image interpretation. No statistical differences were observed (all *p* values > 0.11), except for streak artifacts (*p* = 0.02) which were considered to be more severe, although acceptable, with the 80 kVp protocol.

**Conclusions:**

Reduced tube voltage in combination with IR allows a total iodine dose reduction between 37 and 50%, by using contrast media with low iodine concentrations of 200 and 160 mg I/mL, while maintaining image quality.

## 1. Introduction

Iodine based contrast agents, such as those used in CT and angiography, can cause contrast-induced nephropathy (CIN), which is associated with increased mortality in at-risk patients with renal insufficiency. Several studies demonstrated that CIN incidence is related to the administered iodine load, which motivates the aim for the reduction of the total iodine dose (TID) [1–6]. In CT contrast studies, image quality largely depends on the contrast between enhanced and nonenhanced regions; hence a sufficient amount of contrast agent must be administered to the patient to assure a certain image quality. A reduction of total iodine dose, for example, by reducing the iodine concentration of the contrast media, can only be achieved if the loss of image contrast is compensated. By decreasing the acquisition tube voltage, the attenuation difference between enhanced and nonenhanced tissues increases because the X-ray output energy approaches the iodine k-edge of 33 keV. This increase in image contrast opens up the opportunity to decrease the required iodine dose [7–9]. Scanning at lower photon energies, however, introduces more noise into the images, since X-rays of lower energies are more easily absorbed and consequently less photons reach the detector [10, 11]. This noise increase could be tackled by increasing the tube current, resulting in an increased radiation dose, which is unfavourable considering patient safety. An alternative way to reduce the noise is the use of recently introduced iterative reconstruction (IR) techniques. Instead of using an idealised imaging model like in traditional filtered back-projection (FBP), these IR techniques use an imaging model that describes the image acquisition, including noise statistics, which makes these algorithms more efficient and opens up the possibility of noise reduction [12–17].

Our hypothesis is that the total iodine dose can be substantially reduced when a lower tube voltage of 80 or 100 kVp is used in combination with iterative reconstruction ASiR at a high blending level of 60% in coronary CT angiography.

The impact of the combination of a reduced tube voltage and iterative reconstruction on iodine dose has been the subject of several studies [7, 9, 11, 18], but, to our knowledge, this combination has not previously been reported with contrast media iodine concentrations below 300 mg I/mL for a coronary CT angiography protocol, while still providing images of diagnostic quality.

The objective of this study was to assess the impact of combining a low-tube voltage acquisition with iterative reconstruction in a coronary CT angiography (CCTA) protocol on the necessary iodine dose in a porcine model scanned at constant radiation dose. The specific combinations of contrast media iodine concentration and tube voltage (320 mg I/mL at 120 kVp for the standard of care versus 160 and 200 mg I/mL at, respectively, 80 and 100 kVp for the study protocols) used in this study were selected from previous research in abdominal CT [19–21]. Based on these experiences and on literature [22], we selected a 60% blending level of ASiR reconstruction for this CCTA study.

## 2. Materials and Methods

### 2.1. Porcine Model

Pigs are known to have a similar heart function and blood circulation to humans [23, 24]. Three healthy female, naive Göttingen minipigs (Ellegaard, Dalmose, Denmark) with a mean weight of 40.2 kg (range 38.2–41.5 kg) and a mean effective chest diameter of 24.6 cm (range: 23.9–25.3 cm) were included in the study. Study approval was granted by the institutional ethical committee for animal experiments. Two weeks before the start of the study, a port-a-cath (PAC) unit (Power PAC II, 1.9 mm, Smiths Medical, St Paul, MN, USA) was placed subcutaneously at the level of the left shoulder with a connection to the superior vena cava to allow repeated contrast media injections in a consistent way. The pigs were scanned during a four-month period with an interscan delay of at least 72 hours to avoid iodine retention bias. Anaesthesia was induced by an intramuscular injection of an anaesthetic cocktail (500 mg Zoletil 100, 6.25 mg Rompun, 1.25 mL Ketamine, and 2.5 mL Dolorex) at a dose of 0.05 mL per kg body weight.

### 2.2. Scan and Injection Protocol

Coronary CT angiography (CCTA) was performed in free breathing with heart rate monitoring by a retrospective ECG-gated helical scan, at phase 70–75%, on a 64-slice multidetector CT scanner (Discovery 750HD, GE Healthcare, Waukesha, WI, USA). No medication was used to control the heart rate considering the relatively low and stable rate of mean 62 ± 4 bpm over the total scan period. A standard of care scan protocol was compared with two alternative study protocols (Table 1). The standard of care protocol was scanned at a tube voltage of 120 kVp with standard FBP image reconstruction. Study protocol A was scanned at 80 kVp, whereas protocol B was scanned at 100 kVp. The images of both study protocols were reconstructed with the iterative reconstruction (IR) technique ASiR at 60%. The tube current was adapted for the different scan protocols to result in an equal radiation dose CTDI_vol_ of 28.8 mGy. This was selected based on the exposure conditions of the 80 kVp tube potential scan. The tube current of the other scan sequences was adapted to result in the same CTDI_vol_ value. All scan protocols were acquired with a slice thickness and increment of 0.625 mm and a 30-second scan delay after injection. The scan protocol order was randomly assigned and repeated 3 times on two pigs, with confirmation in a third pig, resulting in a total of 21 scans.

Iodixanol (VISIPAQUE, GE Healthcare, Cork, Ireland) with an iodine concentration of 320 mg I/mL was used as the standard of care contrast media. The reduced iodine concentrations of protocols A (160 mg I/mL) and B (200 mg I/mL) were achieved by formulating the contrast media with additional saline (Baxter, Deerfield, IL, USA). The different contrast media were administered through the port-a-cath unit at controlled room temperature (20°C) using a dual head injector from Nemoto Kyorindo (Tokyo, Japan). Contrast media administration was performed at a constant injection rate (3 mL/s), injection volume (60 mL, ~1.5 mL/kg), and saline chaser (30 mL), resulting in three different iodine delivery rates (IDR) and total iodine doses (TID) (Table 1).

### 2.3. Objective and Subjective Image Quality Parameters

Image quality was objectively evaluated by measuring the average CT value in Hounsfield units (HU) and image noise by the standard deviation (SD) in circular regions of interest (ROIs). These measurements were performed in the proximal coronary segment of the main coronary arteries, the right coronary artery (RCA), the left circumflex artery (LCx), and the left anterior descending artery (LAD), and in the aorta and the heart muscle tissue next to the aortic valve. Coronary ROIs had a diameter of 1.5 mm; the ROIs of the aorta and heart muscle tissue were 10 mm in diameter. The signal-to-noise ratio (SNR) and contrast-to-noise ratio (CNR) were calculated. SNR was calculated as the ratio between the mean CT value and the SD, while CNR was calculated as (mean CT value organ of interest − mean CT value heart muscle)/SD heart muscle.

Two independent blinded expert readers (eight and ten years of experience in cardiac CT) randomly scored the images on four image quality parameters (Table 2) based on reported cardiac CT quality studies [13, 19, 24].

### 2.4. Statistical Analysis

Data were analyzed using commercially available software (SPSS, version 14; SPSS, Chicago, IL, USA). Statistical analysis by a nonparametric Mann–Whitney *U* test was performed to compare the objective measurements (CT signal, SNR, and CNR) of the standard of care protocol with the results of both study protocols. A Chi-square test was used to assess the results of the subjective evaluation by the two expert readers. For both statistical techniques, *p* values less than 0.05 indicate a significant difference. Intrapig and interpig variability was tested by 95% confidence intervals and Mann–Whitney *U* test.

## 3. Results

### 3.1. Objective Image Quality Parameters

**Figure 1:**
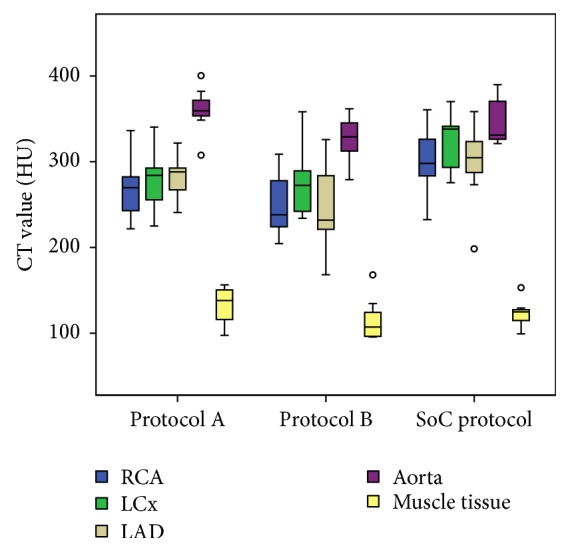
CT values for the right coronary artery (RCA), left circumflex artery (LCx), left anterior descending artery (LAD), aorta, and muscle tissue for both study protocols and the standard of care protocol. Boxplots display the median (middle bar), upper, and lower quartiles, while the whiskers (vertical line) indicate the variability outside the upper and lower quartiles. Mild outliers are displayed as o. No significant differences were found between the CT values of the study protocols and the standard of care protocol for any of the coronary arteries.

**Figure 2:**
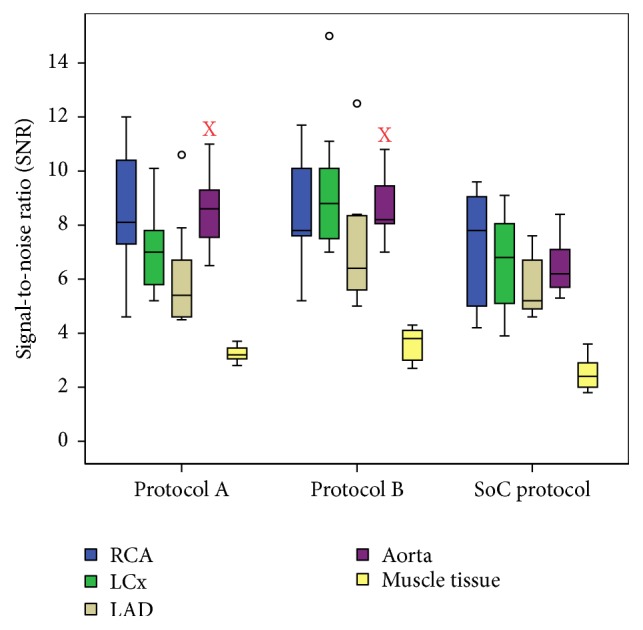
Signal-to-noise ratio for the right coronary artery (RCA), left circumflex artery (LCx), left anterior descending artery (LAD), aorta, and muscle tissue for both study protocols and the standard of care protocol. Boxplots display the median (middle bar), upper, and lower quartiles, while the whiskers (vertical line) indicate the variability outside the upper and lower quartiles. Mild outliers are displayed as o. X indicates that there is a significant difference with the objective parameter of the standard of care protocol.

**Figure 3:**
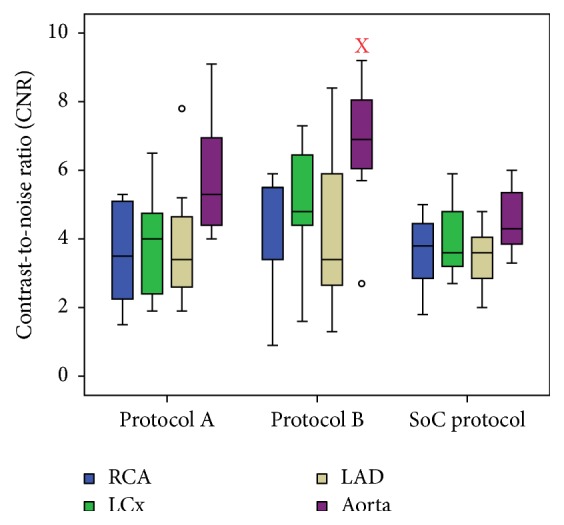
Contrast-to-noise ratio for the right coronary artery (RCA), left circumflex artery (LCx), left anterior descending artery (LAD), and aorta for both study protocols and the standard of care protocol. Boxplots display the median (middle bar), upper, and lower quartiles, while the whiskers (vertical line) indicate the variability outside the upper and lower quartiles. X indicates that there is a significant difference with the objective parameter of the standard of care protocol.

Objective analysis of the data shows no major differences between the mean measured CT values and calculated SNR and CNR of the standard of care protocol and both study protocols (Table 3 and Figures 1–3). The CT signal is slightly but not significantly lower in the coronary arteries after a CCTA with both study protocols as compared to the standard of care acquisition (Figure 1). For example, the mean coronary enhancement of the RCA is 268.3 ± 14.2 HU (*p* = 0.14) for protocol A, 250.7 ± 14.8 HU (*p* = 0.05) for protocol B, and 301.5 ± 15.8 HU for the standard of care protocol. Similar CT values are measured in the other coronary arteries. The SNR and CNR of the study protocols are slightly but not significantly higher compared to the SNR and CNR of the standard of care protocol. For example, the mean SNR of the RCA is 8.6 ± 1 (*p* = 0.48) for protocol A, 8.6 ± 0.8 (*p* = 0.41) for protocol B, and 7.1 ± 0.9 for the standard of care protocol (Figure 2). The mean CNR of the RCA is 3.6 ± 0.6 (*p* = 0.8) for protocol A, 4.3 ± 0.7 (*p* = 0.08) for protocol B, and 3.6 ± 0.5 for the standard of care protocol (Figure 3). Similar results can be observed for the LCx and the LAD. Some significant differences are observed; for example, both study protocols result in significantly higher SNR for the aorta (8.5 ± 0.6 (*p* = 0.02) for protocol A and 8.7 ± 0.5 (*p* = 0.02) for protocol B) compared to the standard of care protocol (6.5 ± 0.5) (Figure 2). The mean aorta CNR of the images from protocol B (6.7 ± 0.8 (*p* = 0.04)) is significantly higher compared to the mean aorta CNR of the standard of care protocol (4.6 ± 0.4) (Figure 3). The soft tissue image noise, measured as the SD of nonenhanced muscle tissue, is significantly lower in the images of protocol B (32.6 ± 2.1 (*p* = 0.004)), scanned with a tube voltage of 100 kVp and with ASiR 60%, compared to the image noise of the standard of care protocol (50.6 ± 3.7) (Table 3). For all three evaluated image quality parameters, no significant differences were observed between both pigs for any of the tested protocols (mean *p* value was 0.24). Also, for the intrapig variability, all obtained results were within the 95% confidence intervals.

### 3.2. Subjective Image Quality Parameters

**Figure 4:**
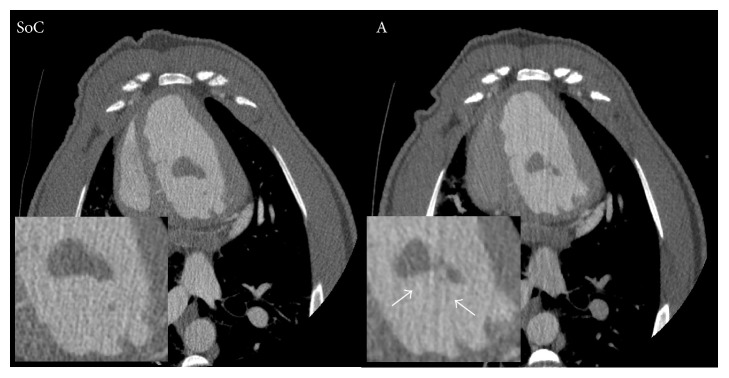
Images of the standard of care (SoC) protocol and study protocol A (A) are compared to demonstrate the significant higher chance on streak artifacts (white arrows) in images scanned at 80 kV (protocol A) compared to 120 kV images (SoC protocol).

**Figure 5:**
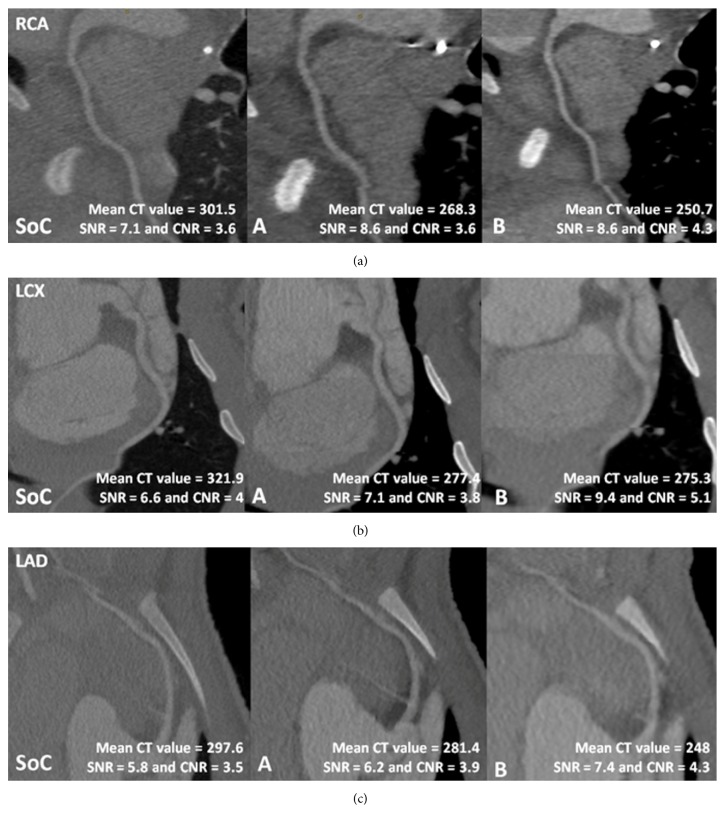
Images of the standard of care protocol and study protocols A and B with the mean CT value, SNR, and CNR of the right coronary artery (RCA) (a), left circumflex artery (LCx) (b), and the left anterior descending artery (LAD) (c).

No significant differences for the overall image quality, vessel sharpness, and image noise are observed between the standard of care protocol and both study protocols. For example, the overall image quality for the standard of care protocol had a median score of 4 (range: 3–5), whereas it was 4 (range: 2–5) for protocol A (*p* = 0.58) and 4 (range: 3–5) for protocol B (*p* = 0.29). Regarding streak artifacts, a significant increase was observed between the standard of care protocol (median score of 4 (range: 2–4)) and protocol A (median score of 3 (range: 2–4) (*p* = 0.021)) (Figure 4) but not with protocol B (median score of 3 (range: 2–4) (*p* = 0.17)). The images of all three scan protocols were evaluated by the observers to be of at least diagnostic image quality on all evaluated parameters (Table 4 and Figure 5).

## 4. Discussion

Several studies have demonstrated that contrast-induced nephropathy (CIN) incidence is related to the administered iodine dose. This motivates the aim for a reduction of the total iodine dose (TID) which can be beneficial for patient safety, especially for patients with a reduced kidney function [1–6].

The total iodine dose used for a coronary CTA, as reported in literature, is typically between 350 and 600 mg I/kg, resulting in a coronary enhancement of 300–400 HU [25–27]. This corresponds well with the total iodine dose (480 mg I/kg) of the standard of care protocol of this study, which uses a contrast media iodine concentration of 320 mg I/mL, resulting in a mean CT signal of 307 HU. Data presented in this study suggest that this concentration can be lowered down to 160 or 200 mg I/mL (total iodine dose of 240 and 300 mg I/kg, resp.), when combined with reduced tube voltages of 80 and 100 kVp, respectively, and additional use of iterative reconstruction ASiR 60%. Even with the reduced total iodine doses of the study protocols, the CT signal, SNR, and CNR were preserved compared to the standard of care CCTA protocol. A noteworthy result is the SNR of the LAD, which was slightly lower compared to the SNR of the RCA and LCx in all three scan protocols. Since the CT signal in the LAD was not inferior compared to the other coronary arteries, it is the slightly higher image noise that is responsible for the lower SNR. A possible explanation is that local noise levels are different in the LAD due to the surrounding tissue or due to the different location of the LAD in the scan field.

The images of both study protocols received equal scores compared to the standard of care protocol's images considering overall image quality, vessel sharpness, and image noise by the expert readers. Streak artifacts were more severe in the images of protocol A in comparison with the images of the standard of care protocol; this appearance of streak artifacts is a well-known observation in low-tube voltage acquisitions [28, 29]. No differences in the appearance of streak artifacts were found between the images of protocol B and the images of the standard of care protocol. This indicates that study protocol B, with a tube voltage of 100 kVp, may be preferred for patients with a larger chest diameter compared to study protocol A, with a tube voltage of 80 kVp, which may be more suitable for thinner patients, because not only the appearance of streak artifacts but also the general image quality of low kV scans is affected by the object size [30]. All images of the three protocols were scored as at least diagnostically acceptable, even with the presence of increased streak artifacts in the images of protocol A which did not impair image interpretation.

From these results, we can conclude that the combination of a reduced tube voltage and the iterative reconstruction ASiR 60% allows a substantial iodine dose reduction in CCTA without compromising diagnostic image quality in a porcine model. An iodine concentration reduction from 320 mg I/mL to 200 or 160 mg I/mL corresponds with an iodine dose reduction in the order of 37% (100 kVp) to 50% (80 kVp). These results are well in agreement with the results described by Nakaura et al. [18] who achieved a similar iodine dose reduction in patients scanned at 80 kVp in combination with a hybrid iterative iDose level of 60% (Phillips Healthcare). The iodine dose reduction, in our study, was achieved by using contrast media with decreased iodine concentrations of 160 or 200 mg I/mL. These are remarkable lower iodine concentrations than typically reported in similar iodine dose reducing studies [7, 9, 11, 18] that used contrast media with iodine concentrations not lower than 300 mg I/mL. These subclinically low iodine concentrations are in strong contrast with the typically higher iodine concentrations used in clinical CCTA practice (between 320 and 400 mg I/mL) [25–27].

As the innovations in iterative reconstruction methods are still ongoing, we expect that more advanced reconstruction methods will result in further noise reduction and hence improved SNR and CNR levels. When SNR and CNR are relevant figures of merit for the diagnostic task, this gain in SNR or CNR could be converted to further reduction of iodine dose. On the other hand, when diagnostic quality is reflected by the CT value (HU) rather than SNR or CNR, further iodine dose reduction will affect diagnostic quality negatively, as iterative reconstruction methods have intrinsically no impact on the CT value. However, in that case, advanced reconstruction methods could compensate for the increased noise levels at lower tube potentials (70 kVp) [31], which could be beneficial for small size patients and children with reduced kidney function [32]. Another beneficial effect of the use of iterative reconstruction methods is the possible reduction of radiation dose exposure, but this was not the aim of this study. Compared to our results, a scan protocol at lower radiation dose would have resulted in less drastic iodine dose reductions for images of equal quality.

For practical reasons, we used a retrospective ECG-gated helical scan technique, which is typically related to a higher radiation dose than a prospective step and shoot technique [33]. This scan technique allowed retrospective manual adjustments of the ECG-based trigger points, which were sometimes suboptimal due to a disturbed ECG signal through the thick pig skin. Nevertheless, similar total iodine dose reduction results can be expected with other cardiac scan techniques.

Limitations of this study are mainly related to the porcine model. Despite the similar cardiac anatomy and function of the minipigs compared to humans, further validation of the alternative study CCTA protocols in patient studies is required before being applied in clinical practice. Although a lot of experimental work to validate new CT technologies can be done in phantom studies, this is not always possible. For example, realistic phantom models that mimic the complex anatomy/physiology of the human heart and coronaries do exist but are rare and are usually confined to investigating a limited amount of physiological parameters [34, 35]. Therefore drastic changes of the scan or injection protocol, like the use of contrast media with subclinical iodine concentrations of 160 or 200 mg I/mL, can be assessed in preclinical animal studies before applying them on patients. The minipigs used in this study were healthy animals with no presence of any coronary stenosis or atherosclerotic plaque. The diagnostic accountability of these pathologies or the ability to analyze the coronary plaque components was not tested in this study. Another limitation is the use of a port-a-cath. The vast majority of CCTA patients have contrast media administered through a peripheral injection in the antecubital vein, while our study used a port-a-cath injection which delivers the contrast media directly to the superior vena cava. This difference in contrast media administration may have an impact on the required iodine dose to result in a sufficient coronary enhancement. However, a similar total iodine dose reduction, by combining a low-tube voltage acquisition with the iterative reconstruction ASiR 60%, may be expected independent of the contrast media administration technique. For example, similar iodine dose reduction results were found in patients with an intravenous contrast media administration [18].

## 5. Conclusion

The results suggest that a significant total iodine dose reduction appears feasible in clinical CCTA by combining low-tube voltage acquisitions with iterative reconstruction techniques, from 480 mg I/kg down to 300–240 mg I/kg or about 37% (100 kVp) to 50% (80 kVp), by using contrast media with iodine concentrations of 200 and 160 mg I/mL, while maintaining image quality as found in this porcine study. Contrast media administration should be continuously reassessed in relation to the evolving CT technology.

## Figures and Tables

**Table 1:** Technical scan and injection parameters of the different scan protocols.

Scan parameters	Standard of care protocol	Protocol A	Protocol B
Tube voltage (kVp)	120	80	100
Tube current (mA)	235	700	375
CTDI_vol_ (mGy)	28.8	28.8	28.8
Scan type	Cardiac helical	Cardiac helical	Cardiac helical
Scan delay (s)	30	30	30
Image reconstruction model	FBP	ASiR 60%	ASiR 60%
Contrast media concentration (mg I/mL)	320	160	200
Injection volume (mL)	60	60	60
Injection rate (mL/s)	3	3	3
Iodine delivery rate (g I/s)	0.96	0.48	0.60
Total iodine dose (mg I/kg)	480	240	300

mg I/mL: milligrams of iodine per milliliter.

g I/s: grams of iodine per second.

mg I/kg: milligrams of iodine per kilogram of body weight.

**Table 2:** Visual score for evaluation of subjective image quality.

Overall image quality (OIQ)	Excellent (5)	Above average (4)	Acceptable (3)	Suboptimal (images still interpretable) (2)	Very poor (nondiagnostic) (1)
Vessel sharpness (VS)	Excellent (5)	Above average (4)	Acceptable (3)	Suboptimal (images still interpretable) (2)	Very poor (nondiagnostic) (1)
Image noise (IN)	Minimal noise (5)	Less than average (4)	Average (3)	Higher than average (2)	Unacceptably high (1)
Streak artifacts (SA)		No artifacts (4)	Mild artifacts (3)	Moderate artifacts but images still interpretable (2)	Severe artifacts hindering the image interpretation (1)

**Table 3:** Mean objective image quality parameters with 95% confidence interval of the three coronary arteries (RCA, LCx, and LAD), aorta, and muscle tissue.

		RCA	LCx	LAD	Aorta	Muscle
SoC protocol	CT value (HU)	301.5 (262.8–340.2)	321.9 (289.5–354.3)	297.6 (250.5–344.7)	347.9 (321.9–373.9)	123.1 (107.6–138.6)
	SD	46.3 (32.1–60.4)	52.9 (37.7–68.2)	53.2 (39.8–66.7)	55.3 (45–65.5)	50.6 (41.6–59.7)
	SNR	7.1 (5–9.2)	6.6 (4.8–8.4)	5.8 (4.7–6.9)	6.5 (5.4–7.6)	2.5 (1.9–3.2)
	CNR	3.6 (2.5–6.1)	4 (3–5.1)	3.5 (2.6–4.3)	4.6 (3.6–5.5)	

Protocol A	CT value (HU)	268.3 (233.5–303.1)	277.4 (242.8–313.1)	281.4 (256.3–306.6)	359.7 (332.9–386.4)	132.1 (110.3–153.8)
	SD	34 (22.8–45.2)	40.8 (32.5–49.1)	50.6 (36.2–65)	42.8 (38.3–47.4)	40.7 (34.2–47.3)
	SNR	8.6 (6.2–11)	7.1 (5.5–8.7)	6.2 (4–8.3)	8.5^*∗*^ (7.1–10)	3.2^*∗*^ (3–3.5)
	CNR	3.6 (2.1–5.1)	3.8 (2.2–5.4)	3.9 (2.1–5.8)	5.9 (4.1–7.7)	

Protocol B	CT value (HU)	250.7 (214.5–287)	275.3 (234.9–315.8)	248 (197.4–298.5)	326.5 (300.8–352.2)	116 (91.1–140.8)
	SD	30.5 (24.1–36.9)	30.7^*∗*^ (24.4–37)	37.1^*∗*^ (22.9–51.3)	37.9^*∗*^ (34.4–41.4)	32.6^*∗*^ (27.5–37.7)
	SNR	8.6 (6.5–10.7)	9.4 (6.8–12)	7.4 (5–9.8)	8.7^*∗*^ (7.5–9.9)	3.6^*∗*^ (3–4.2)
	CNR	4.3 (2.5–6.1)	5.1 (3.3–6.8)	4.3 (2–6.6)	6.7^*∗*^ (4.7–8.7)	

*∗* indicates significant difference compared to reference protocol (*p* < 0.05).

**Table 4:** Median subjective image quality parameters.

	Overall image quality (1–5)	Vessel sharpness (1–5)	Image noise (1–5)	Streak artifacts (1–4)
SoC protocol	4.0	4.0	4.0	4.0
Protocol A	4.0	3.5	3.5	3.0^*∗*^
Protocol B	4.0	4.0	4.0	3.0

*∗* indicates significant difference compared to reference protocol (*p* < 0.05).
